# Transforming growth factor‐β signalling in tumour resistance to the anti‐PD‐(L)1 therapy: Updated

**DOI:** 10.1111/jcmm.17666

**Published:** 2023-01-10

**Authors:** Keywan Mortezaee, Jamal Majidpoor

**Affiliations:** ^1^ Department of Anatomy, School of Medicine Kurdistan University of Medical Sciences Sanandaj Iran; ^2^ Department of Anatomy, School of Medicine, Infectious Diseases Research Center Gonabad University of Medical Sciences Gonabad Iran

**Keywords:** immune checkpoint inhibitor, programmed death‐1 receptor, programmed death‐ligand 1, resistance, transforming growth factor‐β, tumour microenvironment

## Abstract

Low frequency of durable responses in patients treated with immune checkpoint inhibitors (ICIs) demands for taking complementary strategies in order to boost immune responses against cancer. Transforming growth factor‐β (TGF‐β) is a multi‐tasking cytokine that is frequently expressed in tumours and acts as a critical promoter of tumour hallmarks. TGF‐β promotes an immunosuppressive tumour microenvironment (TME) and defines a bypass mechanism to the ICI therapy. A number of cells within the stroma of tumour are influenced from TGF‐β activity. There is also evidence of a relation between TGF‐β with programmed death‐ligand 1 (PD‐L1) expression within TME, and it influences the efficacy of anti‐programmed death‐1 receptor (PD‐1) or anti‐PD‐L1 therapy. Combination of TGF‐β inhibitors with anti‐PD(L)1 has come to the promising outcomes, and clinical trials are under way in order to use agents with bifunctional capacity and fusion proteins for bonding TGF‐β traps with anti‐PD‐L1 antibodies aiming at reinvigorating immune responses and promoting persistent responses against advanced stage cancers, especially tumours with immunologically cold ecosystem.

## 1 INTRODUCTION

Immune checkpoint inhibitor (ICI) therapy using monoclonal antibodies against various checkpoints including programmed death‐1 receptor (PD‐1), programmed death‐ligand 1 (PD‐L1) or cytotoxic T lymphocyte associated antigen‐4 (CTLA‐4) is on the eye of current investigations. The strategy of application of ICIs has revolutionized the field of oncology, but clinical trials attested durable benefits just in a number of patients. Tumour cells take immune escape mechanisms in order to reduce the durability of response to immunotherapy.^1^ Biomarker stratification is a useful tool for assessing ICI efficacy. Tumour mutational burden (TMB) and T‐cell inflamed gene expression profile distinctly capture neoantigenicity and T‐cell activation profile of cancer, so they have low correlation but representing joint predictive utility for identification of responders to immunotherapy.^2^ Adenomas progressed towards colorectal cancer (CRC) are characterized by infiltration of immunosuppressive cells and their co‐localization with tumour cells.^3^ In fact, upregulation of immune checkpoints is presumably co‐occurring with dysregulated cytokine profile in order to promote immunosuppression and tumour metastasis.^4^

Transforming growth factor‐β (TGF‐β) release and PD‐L1 upregulation are the two key contributing factors responsible for immune evasion and tumour aggression. TGF‐β is a multi‐tasking,^5^ an anti‐inflammatory^6^ and a pro‐fibrotic^7^ cytokine that is frequently expressed in tumours^8^ and acts as a key promoter of tumour resistance and metastasis.^9^ High levels of TGF‐β are characteristic of immunosuppressive tumour microenvironment (TME),^10^ particularly in cancers like glioblastoma (GBM),^11^ and are primary mechanism of immune escape in solid tumours.^12^ TGF‐β is, in fact, a tumour‐intrinsic mechanism for evaluation of the functional tumour‐immune state^13^ and investigating responses to ICI therapy. Immune exclusion, non‐inflamed or cold immunity are terms referred to the activity of TGF‐β.^14^ A positive correlation is identified between high stromal TGF‐β with weak prognosis and ICI resistance in lung cancer.^15^ TGF‐β activation, T‐cell exclusion and low TMB are critical hallmarks of microsatellite stability CRC and are indicative of limited response to the ICI therapy.^12^ In gastric cancer, a subtype with overexpression of genes related to the TGF‐β/SMAD pathway also represents lower responses to the anti‐PD‐1 therapy.^16^ Due to the importance of TGF‐β in promoting key tumorigenic events, and its critical contribution to the tumour progression, therapy resistance and metastasis, we aimed to discuss about the impact of this cytokine in regulation of immune checkpoints with a particular focus over PD‐(L)1 checkpoints, which is a hot topic of the current years in the area of cancer therapy. Mechanisms related to the TGF‐β‐mediated bypass of ICI therapy, and the current view over strategies to rescue tumour immunity from the impact of this cytokine in order to improve the efficacy of anti‐PD‐(L)1 are described in this paper.

## 2 PROGRAMMED DEATH RECEPTOR/LIGAND IN CANCER IMMUNITY AND IMMUNOTHERAPY

Checkpoint is regulator of immune responses. Allison and Honjo began ground‐breaking investigations over cancer immunity and immunotherapy in the year 1992, and their discovery was honoured by the 2018 Nobel Prize in physiology and medicine.^17^ Studies further expanded in the area to target a number of advanced stage solid cancers, and the primary results were promising. However, therapy bypass has been a considerable issue of the current years, and many researchers are trying to find ways to hamper this bypassing system and extending the durability of therapy, especially in tumours with cold immunity. Tumoral expression of PD‐L1 is a biomarker associated with therapy response to the ICI therapy.^18^ Single‐cell transcriptome profiling of oesophageal squamous cell carcinoma (ESCC) showed the highest expression of PD‐L1 on tumour dendritic cells (DCs), which is contributed to the T‐cell anergy.^19^ Anti‐PD(L)1 is viewed as an immune normalization approach with higher response‐to‐toxicity profile. Immune normalization vs. immune enhancers is like a big pipeline. Enhancers increase the pressure within the pipeline in order to overcome deficiencies in drainage, but it has a risk of pipeline breakdown when the increased pressure rate is too high. By contrast, the normalizer approach is aimed at restoring the normal flow within the pipeline without risking pipeline breakdown. In the TME, overexpression of PD‐L1 causes overregulation of T cells. Thus, identification of a particular defect, and developing strategies for selective repair of this deficiency and restoring immune competence without causing general immune activation can be a desired anti‐tumour strategy.^20^

## 3 TRANSFORMING GROWTH FACTOR‐Β SIGNALLING IN CANCER

### 3.1 Transforming growth factor‐β regulation

TGF‐β has the high affinity to bind with the TGF‐β receptor II (TGF‐βRII), which further recruits TGF‐βRI into a heterotetrameric complex. The complex initiates SMAD‐related transcriptional repression or activation of genes contributed to the differentiation, growth and migration.^8^ SMAD4 is the key mediator in the TGF‐β pathway; upon activation of TGF‐β, complexes of SMAD are formed through bondage of SMAD2 and SMAD3 with SMAD4. The TGF‐β/SMAD network exerts complex biological activities.^21^ TGF‐β signalling is negatively regulated by SMAD7. In fact, SMAD7 restricts PD‐1‐induced regulatory T‐cell (Treg) differentiation and limits responses from T cells to TGF‐β, which further result in increased intestinal inflammation and progression of colitis. PDL1/2^+^DCs are more developed when they are SMAD7 deficient, such cells induce CD4^+^ T cell‐to‐Treg differentiation and reduce the severity of colitis in mice.^22^ In cancer, a diverse path is taken in which increased activity of TGF‐β and dysregulation in the TGF‐β/SMAD signalling is contributed to tumour initiation and progression in several human cancers.^23^ Tregs suppress T‐cell effector function and dampen anti‐tumour immunity, which can be abrogated after blockade of surface TGF‐β on Tregs.^24^ Beside promotion of canonical SMAD pathway, TGF‐β signalling can take tumour‐promoting activities also through non‐canonical pathways, mediated via interaction with intracellular pathways, such as phosphatidylinositol 3′‐kinase (PI3K)/protein kinase B (AKT) and mitogen‐activated protein kinase (MAPK).^25^

**FIGURE 1:**
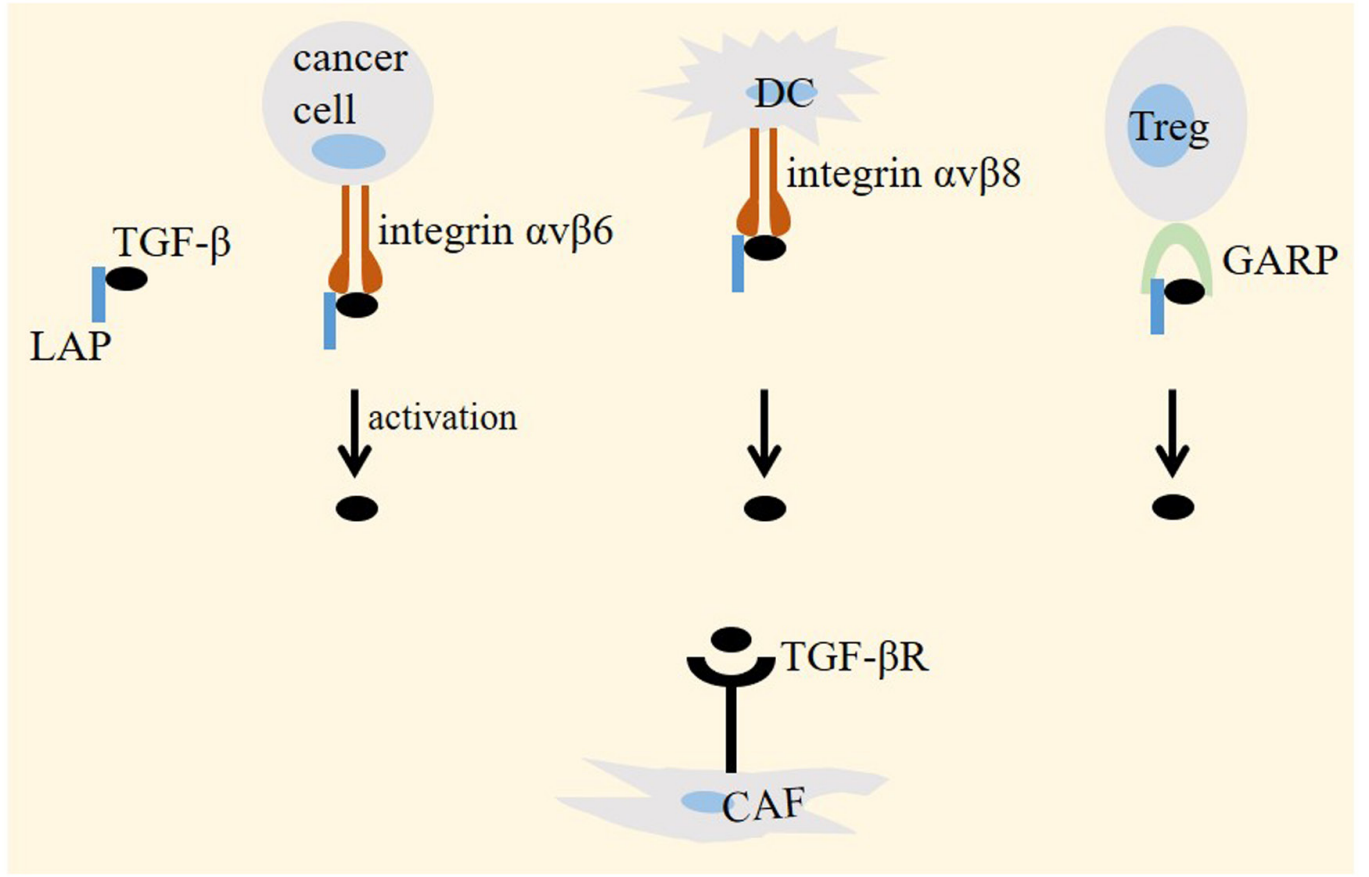
Transforming growth factor‐β (TGF‐β) activation in cancer. TGF‐β is released into the tumour microenvironment (TME) in an inactive form through binding to latency‐associated peptide (LAP). Interaction between the latent TGF‐β with integrin αvβ6 (on cancer cells) or αvβ8 (on dendritic cells/DCs) is contributed to the activation of this cytokine, which acts on its receptor on TME cells, such as cancer‐associated fibroblasts (CAFs). In regulatory T cells (Tregs), bondage between latent TGF‐β with GRAP generates active TGF‐β.

TGF‐β is released in an inactive (or latent) form into the TME. Virtually, latent TGF‐β is secreted from all immune cells, but only some cells are involved in the activation of this cytokine.^26^ Bondage between TGF‐β and latency‐associated peptide (LAP) is dissociated upon interaction with integrin αvβ6 subunit expressed on cancer cells, which results in TGF‐β activation for further reshaping cells within the stroma of tumour.^27^ Targeting LAP stimulates anti‐tumour immunity.^28^ Expression of TGF‐β‐related LAP on surface of myeloid‐derived suppressor cells (MDSCs) is contributed to their increased tolerogenic capacity. LAP^+^ MDSCs stimulate Treg accumulation and suppress T‐cell effector function. Glioma patients show increased expression of LAP on MDSCs. Tolerogenic capacity of LAP^+^ MDSCs is suppressed after TGF‐β blockade, and application of antibody against LAP in such cells slows tumour progression.^29^ In DCs, the integrin αvβ8 subunit is contributed to the activation of latent TGF‐β. TGF‐β1 is also presented on surface of Tregs in an inactive form. Here, TGF‐β1 binds to the GARP. It is found that the membrane protein GARP is contributed to the generation of active TGF‐β1 from Tregs, and antibodies to suppress GARP can block immunosuppressive capacity of Tregs and boost immune responses against infection or cancer. Combination of anti‐GARP antibodies with anti‐PD‐(L)1 improves the efficacy of immunotherapy^26^ (Figure 1).

### 3.2 Transforming growth factor‐β cross‐talking within tumour microenvironment

**FIGURE 2:**
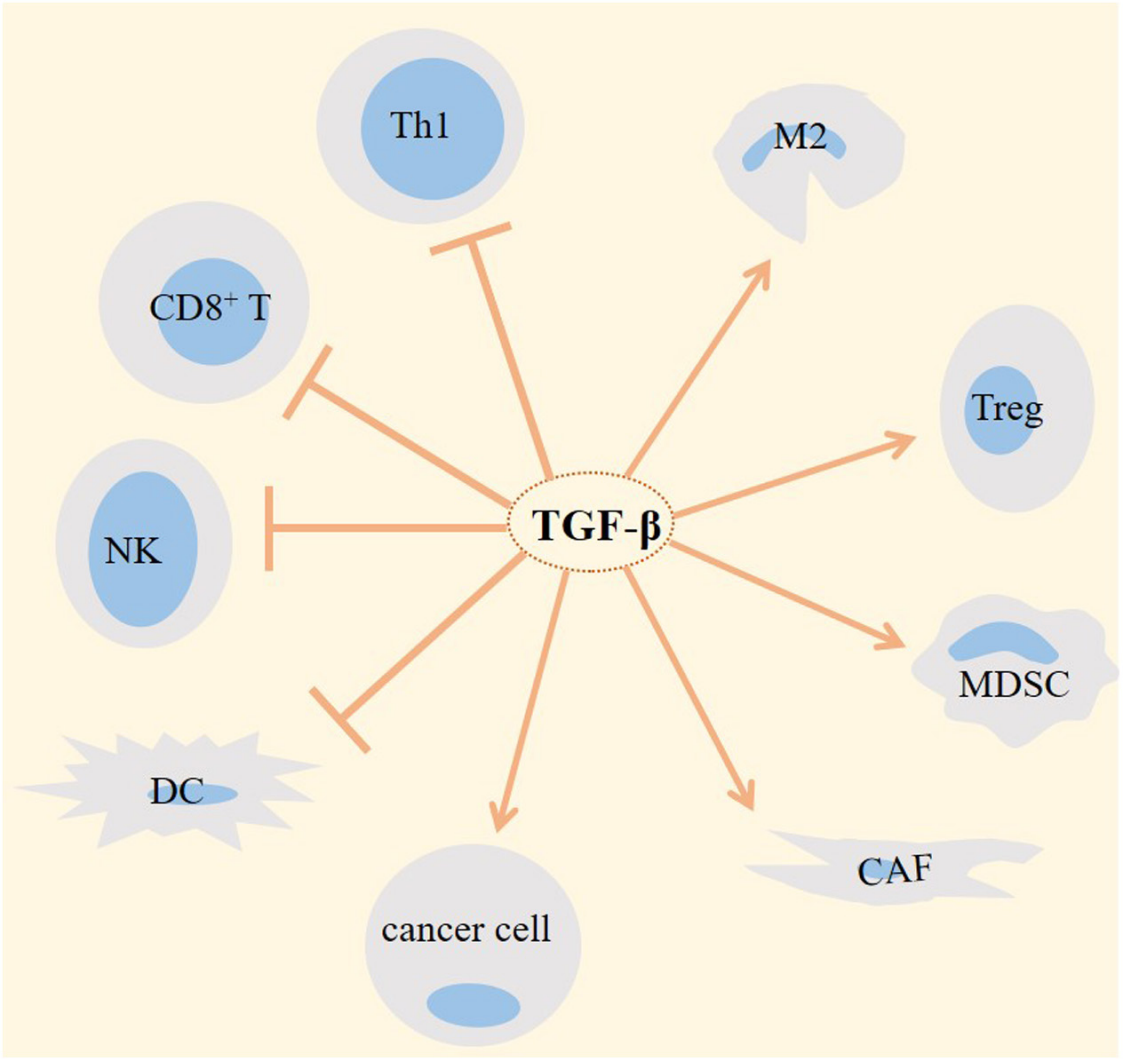
Transforming growth factor‐β (TGF‐β)‐mediated cross‐talking within tumour microenvironment (TME). The activity of TGF‐β is contributed to the progression of tumour through increasing the activity of pro‐tumour cells and dampening the activity of anti‐tumour cells within tumour ecosystem. M2, type 2 macrophage; Treg, regulatory T cell; CAF, cancer‐associated fibroblast; MDSC, myeloid‐derived suppressor cell; NK, natural killer; DC, dendritic cell; and Th1, T helper1.

TGF‐β is secreted from tumour‐associated macrophages (TAMs)^30^ and is activated in tumour endothelial cells (ECs). TGF‐β pathway is pivotal for transition of fibroblasts into myofibroblasts.^19^ Cancer‐associated fibroblasts (CAFs) are one of the critical components of TME that take diverse roles in tumour progression.^31^ Co‐option between CAFs with TAMs causes high secretion of TGF‐β for hindering the effector activity of T cells and inducing generation of Tregs.^32^ High TGF‐β expression from pro‐tumour type 2 macrophages (M2) also inhibits natural killer (NK) cell activity.^33^ Tregs are the cell type abundantly presented in TME of solid tumours.^31^ Induction of Tregs and inhibition of CD8^+^ T and Th1 cells by TGF‐β are contributed to the immune dysfunction within TME.^8^ Myeloid cells activate TGF‐β to promote tumour metastasis.^34^ TGF‐β is placed upstream to the regulation of mammalian target of rapamycin (mTOR) complex 2 (mTORC2). The inducible effect of TGF‐β on mTORC2 promotes invasion of bladder cancer cells^35^; in NK cells, TGF‐β represses mTOR pathway, which results in the suppression of NK cell activation and function^36^ (Figure 2).

## 4 CELLULAR MEDIATORS OF RESISTANCE RELATED TO THE IMPACT OF TRANSFORMING GROWTH FACTOR‐Β ON ANTI‐PD‐(L)1

**FIGURE 3:**
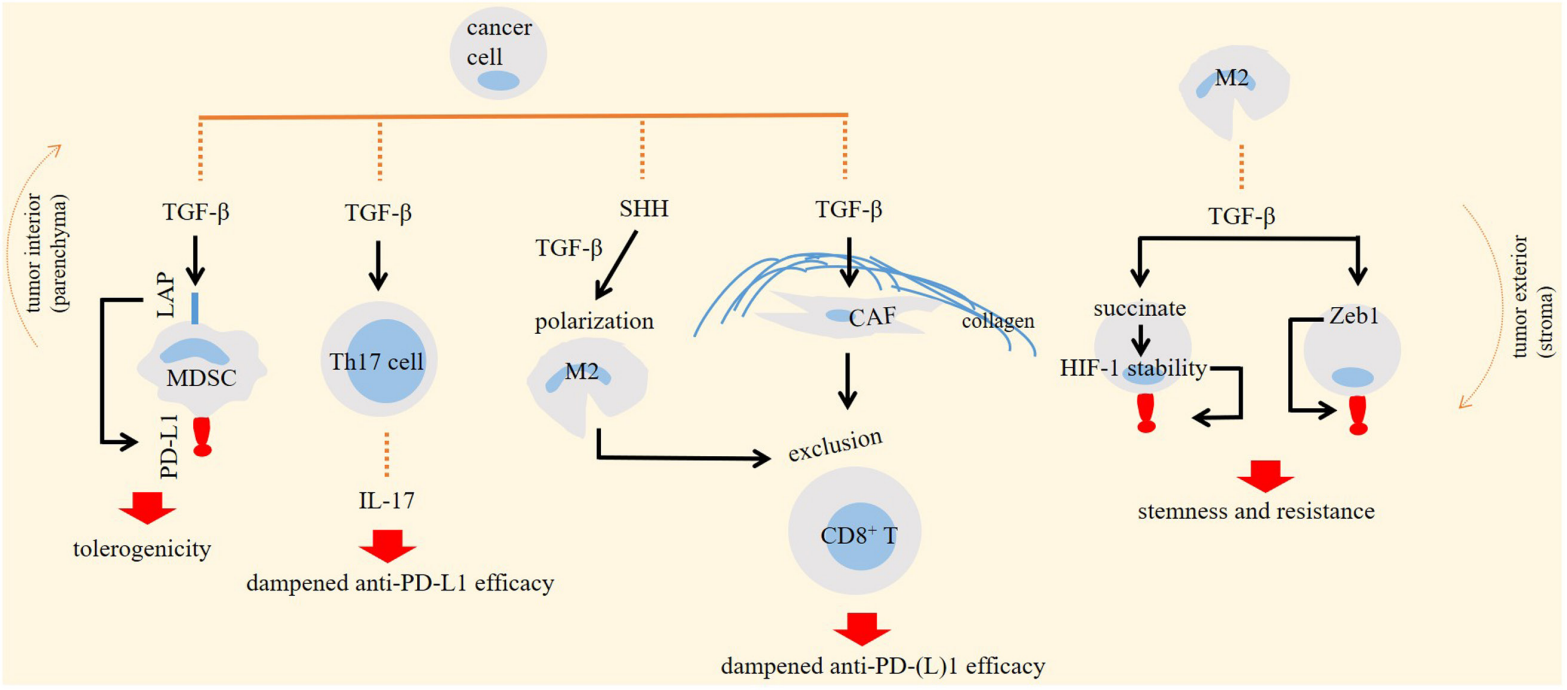
Impact of transforming growth factor‐β (TGF‐β) on cellular tumour ecosystem for promoting resistance to the programmed death‐1 receptor (PD‐1)/programmed death‐ligand 1 (PD‐L1) inhibitor therapy. Enhanced PD‐L1 expression and IL‐17 release, and increased CD8^+^ T‐cell exclusion are mechanisms mediated by TGF‐β for promoting therapy resistance. LAP, latency‐associated peptide; MDSC, myeloid‐derived suppressor cell; M2, macrophage type 2; HIF, hypoxia inducible factor; and CAF, cancer‐associated fibroblast.

TGF‐β promotes resistance to the anti‐PD‐(L)1 through several mechanisms, a summary of which is presented in the Figure 3.

### 4.1 CD8 ^+^ T‐cell exclusion

Immunosuppressive signals, such as TGF‐β hamper persistence of tumour‐reactive T cells in solid tumours.^37^ Anti‐PD‐(L)1 therapy is effective only in a number of patients with metastatic urothelial cancer. Resistance to the PD‐L1 inhibitor atezolizumab is related to the CD8^+^ T‐cell exclusion from tumour interior, instead shifting the cells towards tumour stroma. This is mediated through the inducible effect of TGF‐β on CAFs for constructing a collagenous‐rich stroma.^38^ TGF‐β is among cytokines contributed to the promotion of interactions between SPP1^+^ macrophages with FAP^+^ fibroblasts. This interaction stimulates formation of desmoplastic structures that are immune excluded and restrict the infiltration of T cells. High expression of SPP1 or FAP in CRC patients is contributed to the low efficacy of anti‐PD‐L1 therapy. Conversely, disruption of interactions between the two cell types improve immunotherapy outcomes.^39^

Pancreatic ductal adenocarcinoma (PDAC) displays high PD‐L1 expression, major histocompatibility complex class I (MHC‐I) sequestration, insufficient antigenicity, DC exclusion, and Treg and MDSC attraction, all of which are favouring highly efficient immune evasion profile of such cancer type.^40^ PDAC is the best example of a desmoplastic tumour that secrets high amount of TGF‐β.^41^ In such cancer type, presence of CD8^+^ T cells near to the PDAC cells is indicative of higher overall survival.^42^ Increasing intra‐tumoral infiltration of cytotoxic or effector T cells sensitizes basal‐like mesenchymal PDAC to the anti‐PD‐L1 therapy.^43^ CD8^+^ T‐cell exclusion is promoted by integrin αv‐mediated TGF‐β activation within TME. Targeting αv integrin on lung tumour cells increases the density of CD8^+^ T cells and improves the efficacy of anti‐PD‐1 therapy.^27^ Tumoral infiltration of CD8^+^ T cells is affected from the activity of sympathetic nervous system (SNS), which has harmful and advantageous effects on tumour stroma. In liver cancer model, placing mice in an enrichment environment characterized by social interactions for making the animals happier resulted in a dramatic fall in the expression of cytokines, such as TGF‐β. The environment enrichment also helped to overcome anti‐PD‐1 resistance. This is mediated through involvement of SNS/β‐adrenergic receptors (β‐ARs) signalling for silencing CCL2 and further promoting the infiltration of CD8^+^ T cells.^44^

### 4.2 Th17 cell stimulation

PD‐1^+^ Th17 cells are the population of CD4^+^ T cells that highly express TGF‐β. Co‐culture of PD‐1^+^ Th17 cells with fibroblasts induce production of collagen‐1 and pulmonary fibrosis.^45^ TGF‐β is an inducer of Th17 lineage development.^46^ Treatment of *KRAS* mutant lung cancer with MEK inhibitors induces secretion of cytokines like TGF‐β, which further act for stimulating differentiation of Th17 cells. The cells release IL‐17 for reducing the efficacy of anti‐PD‐L1 therapy.^47^ TGF‐β and IL‐17 are produced from the PD‐1^+^ Th17 cells through increased transcription of signal transducer and activator of transcription 3 (STAT3).^45^

### 4.3 Neutrophil type 2 polarization

TGF‐β within the TME induces polarization of tumour‐associated neutrophils (TANs) towards pro‐tumour N2 phenotype.^48^ N2 neutrophils are contributed to the upregulation of PD‐L1 along with the dysregulation of cytokines in lung environment for promoting immunosuppression and lung metastasis in breast cancer patients.^4^ In gastric cancer, pro‐tumour neutrophils express high levels of PD‐L1 and are contributed to the suppression of T‐cell activity and further progression of cancer, but the cells upregulate PD‐L1 more remarkably compared with cytokines like TGF‐β.^49^

### 4.4 Myeloid‐derived suppressor cell accumulation

Presence of myeloid cells is required for activation of PD‐(L)1 and establishing an immunosuppressive TME in pancreatic cancer.^50^ MDSCs are poorly mature cells that are belonged to the innate immunity, and their accumulation in tumour area confers resistance to the ICI therapy. TGF‐β is released from MDSCs to stimulate the activity of Tregs.^51^ Single‐cell RNA sequencing has shown the impact of S100A9 as a mediator for promoting cross‐talk between tumour and stroma in metastatic tumours.^52^ TGF‐β activity is contributed to the cell surface translocation of S100A9 and its further secretion. S100A9‐ C‐X‐C chemokine ligand 12 (CXCL12) signalling in response to the breast cancer‐associated gene 1 (BRCA1) deficiency induces MDSC accumulation in tumour area and promotes anti‐PD‐1 insensitivity.^53^ Targeting CXCL12 can be used as an approach for rendering synergistic effects with anti‐PD‐L1 in pancreatic cancer.^54^

## 5 TRANSFORMING GROWTH FACTOR‐Β IN CELLULAR IMMATURITY AND ANTI‐PD(L)1 RESPONSES

TGF‐β and PD‐L1 are mediators of immaturity in tumour ecosystem. TGF‐β stimulates inhibitor of differentiation 1 (Id1) that its activity further blocks DC maturation.^55^ Under the influence of TGF‐β, LAP is expressed on immature DCs and is contributed to the suppression of T‐cell activation.^56^ TGF‐β also hampers NK cell maturity and further promotes tumour progression. In addition, elevated TGF‐β level suppresses the differentiation program in naive T cells, thereby reducing the number of Th1 effector cells.^55^ Haematopoietic stem cells (HSCs) are CD38^−^ and transcriptomatically immature. High presence of HSCs in the GBM samples is positively associated with increased TGF‐β1 and PD‐L1 levels and supports immunosuppression and malignancy of such cancer type.^11^

Epithelial‐mesenchymal transition (EMT) is a required process during embryo development and at the time of tissue repair, but it confers as a feature of malignant profile^57^ and a promoter of tumour aggression in cancer.^58^ EMT is required for invasion of tumour cells,^59^ but is repressed in the metastatic colonization step, indicated by the reduced EMT inducer Prrx1.^60^ Zeb,^61^ Snail and Slug (Snail2) are transcription factors related to the EMT profile in tumour cells.^62^ The activity of TGF‐β/SMAD is critical for promotion towards acquisition of EMT and metastasis.^21^, ^63^ TGF‐β is released from TAMs,^30^ and there is a positive cross‐talk between TAMs with mesenchymal‐like tumour cells for promotion of metastasis of breast^64^ and CRC cells.^65^ Chronic exposure to the TGF‐β promotes a stable EMT state in mammary carcinoma cells accompanied by stable generation of cancer stem cells (CSCs) and drug resistance that cannot be reversed after withdrawal of TGF‐β, but it is responsive to the mTOR inhibition.^66^ Beside tumour cells, CAFs also show a signature of EMT profile.^67^ Metastatic renal cancer samples are enriched in TGF‐β and EMT signalling.^59^ ESCC is equipped with CST1^+^ myofibroblasts that are characterized by high EMT profile and TGF‐β pathway. In such tumour type, interaction score for the CTLA4‐CD80/86 and PD1‐PDL1 (CD274) is higher in tumour samples compared with normal counterpart.^68^ Zeb1 is required for expression of PD‐L1 on invading lung cancer cells,^69^ but there are tumours that show high EMT but low PD‐L1 expression profile. Enriched TGF‐β signalling and EMT is a characteristic of mesenchymal and basal‐like 2 (BL2) subtypes of triple‐negative breast cancer (TNBC). The mesenchymal subtype shows low expression of PD‐L1 along with suppression of MHC‐I and displays resistance to many compounds, but represents a level of sensitivity to the TGF‐βRI suppressor SB‐505124.^70^ Dual targeting of TGF‐β/PD‐L1 is presumed to hamper EMT/stemness, tumour aggression and resistance.^71^ Whether it is also applicable in tumours under chronic stimulation with TGF‐β is an open question, requiring further illustrations.

## 6 TRANSFORMING GROWTH FACTOR‐Β IN TUMOUR METABOLISM, HYPOXIA AND CHECKPOINT REGULATION

Metabolic reprogramming is a characteristic hallmark of solid tumours.^72^ Engagement between TAM‐derived TGF‐β with its receptor on breast cancer cells inhibits STAT1 transcription and reduces succinate dehydrogenase, which result in the accumulation of succinate in tumour cells. High succinate content increases the stability of hypoxia inducible factor‐1 (HIF‐1) and the subsequent shift in tumour cell metabolism towards glycolysis. HIF‐1 enhances vascularization and increases the expression of PD‐L1 on tumour cells, thereby increasing immunosuppressive activity of tumour.^73^ Hypoxia is a state of O_2_ low condition developed as an adaptive response within the TME^74^ and acts as a promoter of immunosuppression.^75^ Hampering hypoxia sensitizes tumour to therapy.^76^ The activity of TGF‐β is induced under hypoxia,^77^ and that suppression of TGF‐β attenuates the level of hypoxia in tumour area.^41^ A point here is that, TGF‐β signalling stimulates angiogenesis in tumours, mediated through increased expression of vascular endothelial growth factor (VEGF),^5^ and an increase in the tumour vasculature will finally boost hypoxia due to being aberrant and leaky.^75^ These are indicative of a bi‐directional association between TGF‐β with increased hypoxia in tumour ecosystem. A point of notice here is that short‐term and long‐term hypoxia promote reversible and perpetual EMT, respectively, with the latter depended on the activity of HIF‐1.^78^ Besides, chronic TGF‐β exposure is contributed to the chronic hypoxia.^5^ Thus, relation between hypoxia with increased TGF‐β signalling and EMT state of tumour is predictable, and it seems that such interrelations finally promote PD‐L1 upregulation in tumour area, as it also attested a positive correlation between HIF‐1 activity with increased PD‐L1 expression on tumour cells.^73^ Glycogen synthase kinase 3β (GSK3β) is a metabolic enzyme and a tumour suppressor that its activity is hampered under hypoxic conditions of TME.^79^ TGF‐β activates PI3K/AKT,^25^ and the activity of AKT suppresses GSK3β. Activated GSK3β augments the degradation of PD‐L1, thereby reducing PD‐L1 level.^80^

## 7 FROM THERAPEUTIC STANDPOINT

TGF‐β signalling can be directly or indirectly targeted by a number of strategies applied so for boosting the efficacy of immune system against tumours of solid organs. Anti‐PD‐1^81^ and anti‐TGF‐β^82^ can be used synergistically with irradiation for promoting anti‐tumour immunity and hampering tumour metastasis. Repression of TGF‐β, tumour fibrosis and immunosuppressive state can be a target of combined CXCR4 blockade with ICI therapy^83^, ^84^ and other combinatory agents. Nanoparticles or nanodrugs can be used for improving the activity of immune system and increasing the efficacy of ICI.^85^ pH‐sensitive nanodrugs loaded with curcumin, the natural extract with strong immunomodulatory activity,^58^ and anti‐PD‐1 can be used effectively in this context. The nanodrug system is delivered into the TME through bonding to the PD‐1^+^ T cells, and release its contents in acidic TME. The anti‐PD‐1 released from the nanodrug blocks PD‐1 on T cells. Within the TME, curcumin is selectively taken up by TAMs and tumour cells, and suppresses NF‐kB pathway and TGF‐β secretion from the cells and reduces PD‐L1 expression.^86^ Combination of TGF‐β inhibition with the bispecific glucocorticoid‐induced tumour necrosis factor receptor–related protein (GITR) ligand/anti‐PD‐L1 molecule augments the efficacy of therapy in tumours with immune‐excluded ecosystem. GITR is a potent inducer of T‐cell effector function, and the addition of anti‐TGF‐β hampers the immune suppressor activity of Tregs.^87^ Due to the importance of this cytokine in TME immunosuppressive state, it can even be considered as a marker of response prediction to therapy. Strategies to hamper TGF‐β activity and regulate immune checkpoint expression in cancer are presented below.

### 7.1 Targeting lysine specific demethylase 1

Growth and differentiation factor 1 (GDF1) is the member of TGF‐β superfamily that shows high expression profile in weakly differentiated high‐grade hepatocellular carcinoma (HCC). GDF1 induces a dedifferentiation program in HCC cells and increases tumour cell dissemination and metastasis. GDF1 acts through ALK7–SMAD2/3 and suppresses lysine specific demethylase 1 (LSD1) to reactivate cancer testis antigens (CTAs). CTAs stimulate HCC immunogenicity. LSD1 ablation may sensitize GDF1^+^ HCC patients to the anti‐PD‐1 therapy.^88^ In tumours with cold immunity, ablation of LSD1 enhances T‐cell infiltration and tumour immunogenicity and increases responses to anti‐PD‐1 in ICI refractory tumours.^89^

### 7.2 Targeting hedgehog signalling

Presence of cancer‐associated mesenchymal stem cells (CA‐MSCs) in a tumour is closely associated with CD8^+^ T‐cell exclusion in both human and mice. In mouse model of ovarian cancer with hot immunity, CA‐MSCs are negatively correlated with the number of intra‐tumoral CD8^+^ T cells and responses to the anti‐PD‐L1 therapy, and this is mediated through secretion of chemokines and cytokines, such as TGF‐β. Such effects were found to be counteracted after treatment with hedgehog inhibitors, so targeting this signalling in CA‐MSCs can reduce the expression of TGF‐β, turn the tumour‐immune ecosystem into hot, and restore responses to the anti‐PD‐L1 therapy.^33^ Sonic hedgehog (SHH) is a hedgehog ligand that its secretion from tumour cells is contributed to the polarization of macrophages towards pro‐tumour TGF‐β ^high^ M2 phenotype, which further act for exclusion of CD8^+^ T cells. Combination of the hedgehog inhibitor vismodegib with anti‐PD‐1 synergistically reduced tumour growth in mice model of hepatoma and lung carcinoma.^90^

### 7.3 Zebularine‐PPD combination

Combination of gene therapy with epigenetic regulation is an effective strategy for relieving tumour‐immune escape. Combination of the DNA methyltransferase inhibitor Zebularine with the mPEG‐b‐PLG/PEI‐RT3/DNA (PPD) can be used for this purpose. Zebularine is for enhancing the capacity of antigen presenting machinery through inducing the expression of MHC‐I and stimulating DC maturation. PPD is a strategy for delivery of plasmid‐encoding shPD‐L1 in order to downregulate the expression of PD‐L1 on tumour cells, thereby retaining the functionality of anti‐tumour T cells. Zebularine/PPD combinational therapy downregulates the level of TGF‐β and PD‐L1, reduces the proportion of pro‐tumour immune cells (M2, Tregs and MDSCs), suppresses tumour growth and relapse, and prevents metastasis.^1^

### 7.4 Combined TGF‐β/PD‐L1 blockade therapy

**FIGURE 4:**
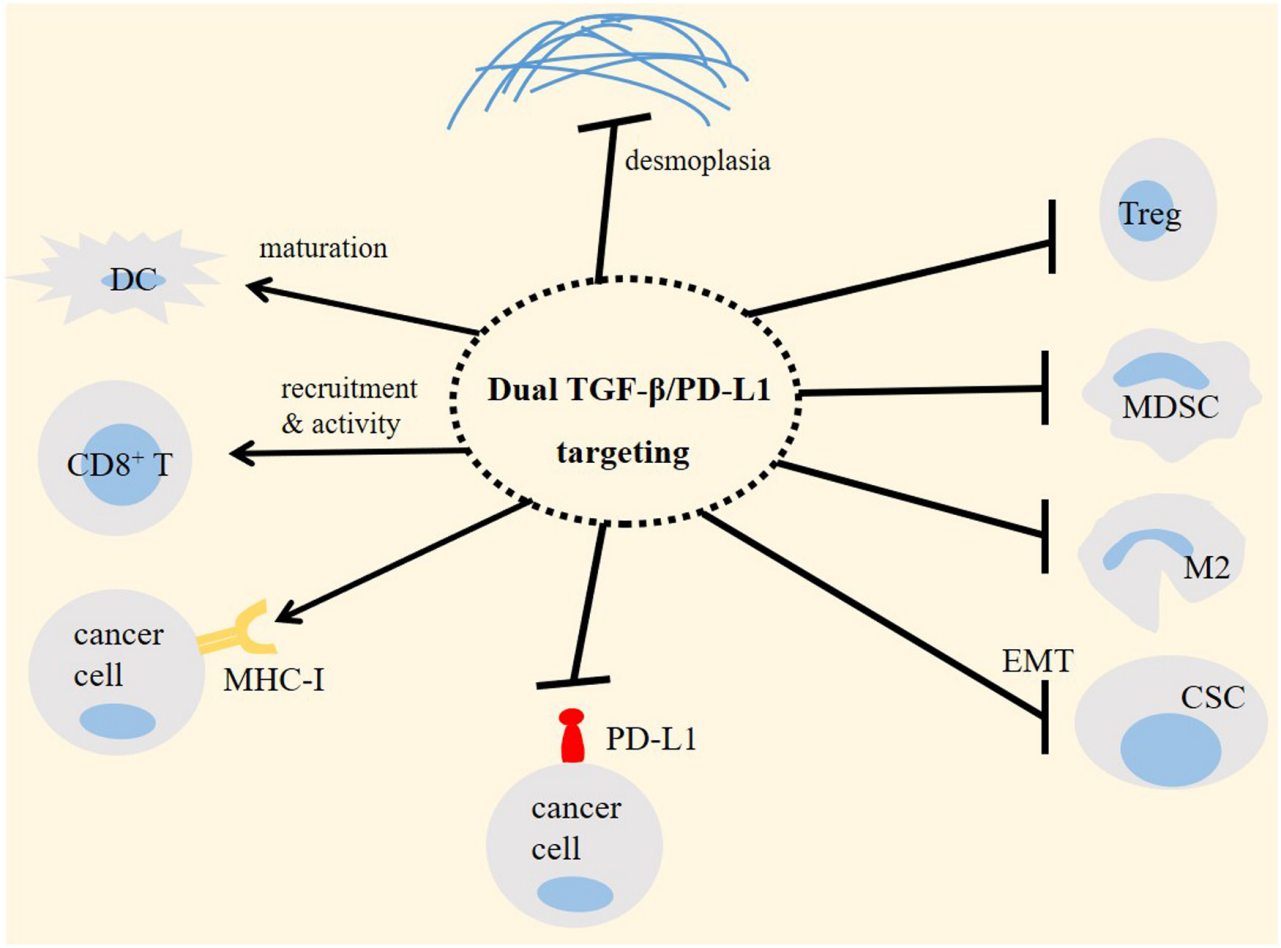
Efficacy of dual targeting of transforming growth factor‐β (TGF‐β)/programmed death‐ligand 1 (PD‐L1) in cancer immunotherapy. Dual TGF‐β/PD‐L1 targeting using bifunctional fusion proteins, such as M7824 (bintrafusp alfa) is a promising and an effective approach for reinstating immune‐permissive profile in tumour ecosystem and boosting the efficacy of immunotherapy. Treg, regulatory T cell; MDSC, myeloid‐derived suppressor cell; M2, macrophage type 2; CSC, cancer stem cell; DC, dendritic cell; MHC‐I, major histocompatibility complex class 1 (MHC‐I); and EMT, epithelial‐mesenchymal transition.

Targeting TGF‐β pathway is a promising approach in cancer immunotherapy.^91^ Targeting TGF‐β in mice with liver metastasis rendered tumour sensitivity to the PD‐(L)1 inhibitor therapy.^12^ Antibodies against TGF‐β and PD‐L1 co‐administered in immune‐excluded mammary mouse model enhanced T‐cell penetration towards tumour interior and reduced tumour burden.^38^ Y‐traps are antibody‐ligand traps that contain an antibody against PD‐L1 or CTLA‐4 fused to an ectodomain sequence of TGF‐βRII, which suppresses both autocrine and paracrine TGF‐β signalling in TME. The efficacy of anti‐CTLA‐4 TGF‐βRIIecd in reducing Treg activity in TME and hampering tumour progression is superior to that for the CTLA‐4 inhibitor ipilimumab. Similarly, the efficacy of anti‐PD‐L1 TGF‐βRIIecd is higher compared to that for the PD‐L1 inhibitors avelumab or atezolizumab.^8^ Bifunctional fusion proteins can be designed in this way for rendering dual anti‐immunosuppressive activity and eliciting durable protective effects (Figure 4). M7824 (also called Bintrafusp alfa or MSB0011359C) is an example of bifunctional agents for boosting anti‐tumour immunity. M7824 includes fully human monoclonal antibody against PD‐L1 (avelumab) attached from its C‐terminus to the two extracellular domain of human TGF‐βRII, with the latter acting as a TGF‐β ‘trap’ for all TGF‐β isoforms. M7824 hampers interactions between PD‐L1 and PD‐1 and simultaneously attenuates or eliminates TGF‐β from the TME.^92^ In fact, suppression of TGF‐β will further boost the efficacy of anti‐PD‐L1 for tumour eradication in which responders to the M7824 therapy display a shift in the tumour ecosystem into a more immune‐permissive profile, as attested by single‐cell RNA sequencing of SCC.^93^ M7824 activates responses from innate and adaptive immunity, hampers tumour growth and metastasis, and exerts superior anti‐tumour activity compared to the monotherapy with either of the agents.^94^ Compared to the anti‐PD‐L1, M7824 maintains the antibody‐mediated anti‐tumour capacity, increases expression of molecules contributed to the trafficking of T cells within tumour area, boosts tumour lytic activity of antigen‐specific CD8^+^ T cells and enhances tumour cell lytic activity of TRAIL.^92^

M7824 can be used as an effective strategy for reverting mesenchymalization in human lung cancer cells,^95^ thereby reducing the invasive behaviour of such cancer and dampening the possibility of tumour resistance and recurrence. Treatment with M7824 also promotes activation of NK and CD8^+^ T cells, and its combination with cancer vaccines rendered superior anti‐tumour efficacy compared with that for anti‐PD‐L1 or TGF‐β targeted agents.^96^ Combination of M7824 with localized radiotherapy in animal tumour model with compromised immunity reprogrammed TME for overcoming immune evasion and attenuated radiation‐mediated lung fibrosis.^97^ Phase 1 clinical trial (NCT02517398) confirms safety and efficacy of M7824 in patients with advanced solid cancers. Grade 3 adverse events are reported in 3/16 heavily pretreated cases. M7824 at different doses shows evidence of efficacy. Administration of 2 doses of this agent reduced the lesion diameter by 25%. Preliminary signs of efficacy related to M7824 warrant future investigations for better identification of its pharmacokinetics, immunogenicity and efficacy.^98^ Data collected from serum concentration in non‐small cell lung cancer (NSCLC) patients treated with 500 and 1200 mg of M7824 (Q2W) and further analysis of overall response rate (per investigator) and progression‐free survival (Kaplan–Meier analyses) further support the application of 1200 mg (Q2W) as the dose recommended for M7824 in phase 2 trial. Responses in patients receiving 500 mg (the lowest exposure quartile) and 1200 mg were 10 and 25–30% (for AUC), respectively.^99^

TGF‐β is overexpressed considerably in patients with human papillomavirus (HPV)^+^ cancers. In a phase 1 trial (NCT02517398), clinical efficacy of different doses of M7824 was compared in patients with HPV^+^ solid cancers (11 from 16 patients had known HPV^+^ disease). Confirmed responses were seen in 45.5% (5/11) of cases, and disease reduction was in 56% (9/16). Grade 3 adverse events were identified in 3/16 cases, and the only grade 4 adverse event was hypokalaemia.^100^ The respective confirmed objective response and disease control rate of 33.5% and 44.1% are also reported in HPV‐associated cancers (results from phase 1 [NCT02517398] and phase 2 clinical trials [NCT03427411]).^101^ Patients with advanced head and neck squamous cell carcinoma (HNSCC) who progressed after platinum‐based therapy and heavily pretreated with M7824 (1200 mg, Q2W) showed a confirmed objective response rate of 13% per independent review committee (16% per investigator), which was more pronounced in HPV^+^ compared with PD‐L1^−/+^ patients. Grade 3 adverse events were identified in 34% of patients, but there were no grade 4 adverse events (NCT02517398).^102^ Analysis of peripheral immunome in HPV‐associated advanced cancer patients showed that in cases who responded to the M7824, high proportion of HPV‐specific CD8^+^ T cells, low neutrophil/lymphocyte ratio and decreased level of IL‐8 were identified (NCT02517398 & NCT03427411).^103^

## 8 CONCLUSIONS

From what discussed above, it could be asserted that the activity if TGF‐β is important for dampening tumour responses to the anti‐PD(L)1 therapy. Thus, reinstating the activity of anti‐tumour immunity using TGF‐β inhibitors can be an effective strategy for improving the efficacy and durability of ICI therapy. A suggested strategy is to use fusion proteins or peptides for targeting both TGF‐β signalling and PD‐L1, which is the current focus in cancer immunotherapy. PD‐L1 expression, however, shows considerable heterogeneity among tumours, which indicates the essence of the utility of indication‐specific strategies to formulate treatment approaches. Besides, the upregulation of alternative checkpoints^104^ and implication of non‐immune pathways to the ICI outcomes also require attention.^105^ Another point to consider is that although TGF‐β is a critical driver of immunosuppression in TME, it is not the solo factor in this context. There are other cytokines and chemokine that implement such activity either in relation with or independent on TGF‐β activity, and their effects are different from one tumour to another. This indicates that the inspection towards the microenvironment of tumour must be tumour‐type specific, and therapeutic regimen must be designed based on considering dominant drivers of immune evasion in the TME of each tumour type. Gathering more information about interactions between cells and driver signalling pathways in tumour stroma will be without a doubt outstanding in order to choose the best therapy or combinatory agent and to improve the idea of personalized therapy.

## 9 FUTURE DIRECTIONS

Understanding more about epigenetic regulators of TGF‐β pathway in tumours and developing strategies to interfere with their expression profile can be effective for improving responses to ICI therapy. Histone demethylases can be a focus in this context.^106^ Beside the applicability in ICI therapy, targeting TGF‐β can also be effective in adoptive T‐cell therapy, which is the current focus in cancer immunotherapy. Chimeric antigen receptor (CAR) T cells armoured against TGF‐β can be developed as a feasible and safe strategy for this purpose.^10^ Another point is that due to the effect of psychological stress on TGF‐β activity and responses to the anti‐PD‐(L)1^44^ and limited studies about possible contribution of stressors in different types of solid cancers, it is suggested to have more focus in ongoing research in this area.

## AUTHOR CONTRIBUTIONS

**Jamal Majidpoor:** Investigation (equal); writing – original draft (equal); writing – review and editing (equal). **Keywan Mortezaee:** Conceptualization (equal); writing – review and editing (equal).

## CONFLICT OF INTEREST

The authors declare that they have no conflict of interest.

## Data Availability

Not applicable.
